# Author Correction: Challenges in implementing cultural adaptations of digital health interventions

**DOI:** 10.1038/s43856-024-00479-x

**Published:** 2024-03-20

**Authors:** Vasileios Nittas, Paola Daniore, Sarah J. Chavez, Tyler B. Wray

**Affiliations:** 1 Department of Behavioral and Social Sciences, Brown University School of Public Health, 121 South Main Street, 02912 Providence, RI USA; 2 Institute for Implementation Science in Health Care, University of Zurich Faculty of Medicine, Universitaetstrasse 84, 8006 Zurich, Switzerland; 3 Center for Alcohol and Addiction Studies, Brown University School of Public Health, 121 South Main Street, 02912 Providence, RI USA

**Keywords:** Public health, Medical research

Correction to: *Communications Medicine* 10.1038/s43856-023-00426-2, published online 05 January 2024

In this article the grant number 202914 relating to Swiss National Science Foundation for V.N. was omitted. The original article has been corrected.

